# Pashtu Language Digits Dataset

**DOI:** 10.1016/j.dib.2022.108701

**Published:** 2022-10-26

**Authors:** Rehan Ullah Khan, Khalil Khan

**Affiliations:** a Department of Information Technology, College of Computer, Qassim University, Buraydah, Saudi Arabia; b Department of Information Technology and Computer Science, Pak-Austria Fachhochschule, Institute of Applied Sciences and Technology, Pakistan; c Faculty of Computer Sciences and Information Technology, Superior University, Lahore, Pakistan

**Keywords:** Natural language processing, Text recognition, Machine learning, Optical character recognition, Pashtu Language Digits Dataset, PLDD, Natural Language Processing, NLP, Machine Learning, ML

## Abstract

Pashtu is a language spoken by 50 million people in the world [1]. It is the national language of Afghanistan and also spoken in the two largest provinces of Pakistan. It is a language written in complex way by calligraphers. Instead of enormous literature and research work in Optical Character Recognition for other languages of the world, this language still requires a mature optical character recognition system [2], [3]. A real dataset of Pashtu digits having 50000 scanned images is introduced and made publically available in this paper. All the digits in the images are handwritten images written and collected from faculty members, staff, and students of the Pak-Austria Fachhochschule, Institute of Applied Sciences and Technology, Pakistan. A total of 1250 candidates appeared in writing the text, out of which half are male and half female. The dataset will be publically available for research purposes.

**Specifications Table**

**Table:** 

Subject area	Computer science, Signal Processing
Specific subject area	Image processing, optical character recognition, Pashtu language digits recognition
Type of data	Images
How data was acquired	Original data are collected through hand written text. These texts are written by the faculty members and students of the Pak-Austria Fachhochschule, Institute of Applied Sciences and Technology, Pakistan. After writing, the text images are scanned at 300 DPI.
Data format	Raw
*Description of data collection*	To keep diversity in writing style, we collected data from different people belonging to different regions. All digits are handwritten images collected from faculty, staff, and students of the institute. A total of 1250 participants were involved in the writing task, out of which half were male and half female. All participants were given a consent form that the dataset would be published and used for research purposes and scientific use. Each participant wrote each numeral four times, including digits from 0 to 9. One sheet per person contributes 40 digits. We scanned all images as greyscale and then converted them into binary form. To make the dataset diverse, no participant was again given the sheet for writing.
Data source location	Pak-Austria Fachhochschule, Institute of Applied Sciences and Technology, Pakistan
Experimental factors	We ask 1250 participants to write all 10 digits (0-9) of Pashtu language in a paper given to them. All images are scanned with 300 DPI.
Data accessibility	We named our proposed dataset as Pashtu Language Digits Dataset, abbreviated as PLDD. Link to the proposed dataset is https://data.mendeley.com/datasets/zbyc7sgp63/1. To access the dataset doi: 10.17632/zbyc7sgp63.1 can be approached.

## Value of the Data

• The data is valuable for the field of Computer Vision and Image Processing, especially for the Pashtu language text recognition. *The PLDD is of interest to researchers, natural language processing (NLP) experts, and industrial experts working on NLP.*
• The data provided can be used to train a machine learning based models to be used for OCR system of Pashtu language. Pashtu OCR is still an open research area. This database is part of our research strategy for long term which will eventually reach to a mature OCR for Pashtu language.
• The database can help researchers and practitioners to build OCR system for Pashtu language. The data is also useful as a reference dataset for benchmarking model.

## 1 Data Description

**Fig. 1:**
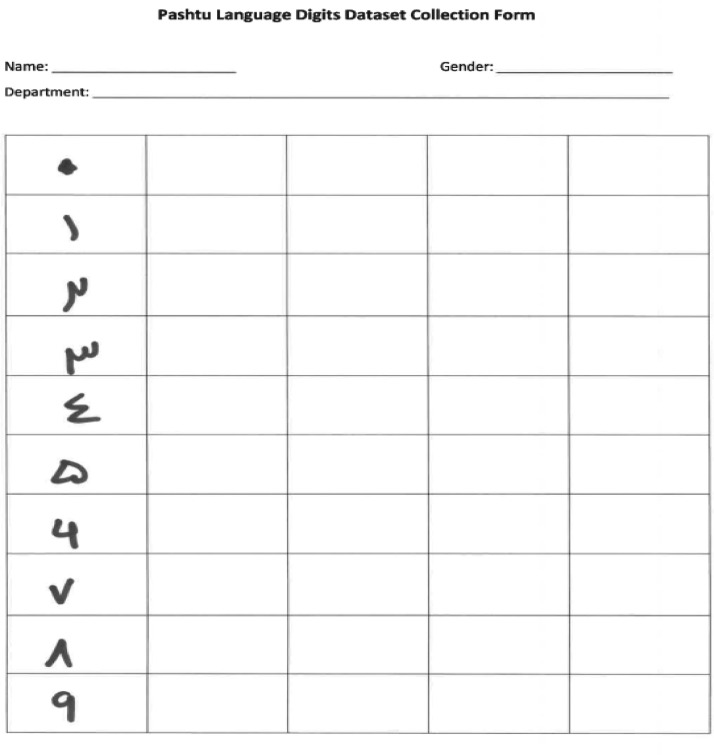
Sample form distributed among students, staff, and faculty members.

PLDD is the Pashtu handwritten digits database. Specific forms on a format as shown in Fig. 1 were distributed among students, faculty members, and staff of the the Pak-Austria Fachhochschule, Institute of Applied Sciences and Technology, Pakistan. All participants were requested to fill these forms through handwritten text. For later on usage of the database, gender equality was considered while writing the text. Half of the participant were male and half female. After collected of the forms, all images were scanned in RGB form. After some pre-processing data was converted into binary form. Hence, all images in the PLDD are binary images. PLDD contains 50000 scanned images.

## 2 Experimental Design, Materials and Methods

We ask 1250 people, including faculty members and students of the Pak-Austria Fachhochschule, Institute of Applied Sciences and Technology, Pakistan, to write all ten digits of the Pashtu language on a plain sheet of paper. Fifty percent of the subjects are male and 50 % female. Participants in the proposed database have age range between 18-60 years. We scanned each page with a 300 DPI. A sample shown in Fig. 1 shows the form distributed amoing faculty, students, and staff.

**Fig. 2:**
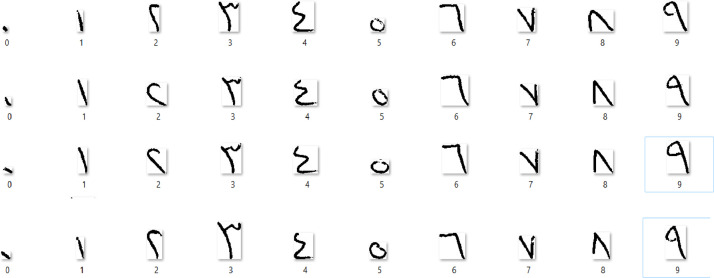
Pre-processed PLDD images.

After scanning, all ten digits are extracted from the images with the following steps:

• Currently available most of the optical character recognition systems read linear form of text only. These methods have limitations while reading artistic and somehow nonlinear text. To remove all these deficiencies inclination of the text is corrected. We also use text inclination method [4], [5], [6]. We corrected the inclination of the scanned page using horizontal histogram [6].
• In most of the center labeling algorithm the provisional labels usually propagate in a definite direction on the connected components. An algorithm has been proposed in [7] in which a single dimensional table also called connection table memorized various labels equivalences during operations. These labels propagate not only on the connected components but also on the table. By this way the connectivity between labels (provisional) at some geometric distance is reflected on the propagation of labels. This process normally reduces the number of scans performed. In the method proposed in [7] both backward and forward scan are performed successively through the label connection table. This way the labeling process is very fast. In the pre-processing stage we detected the center of every digit by the same way i.e., connected component method [7].
• Each page contains all ten digits (0-9), while each digit is written four times (Fig. 1) on paper by each candidate. In the proposed pre-processing stage, we extracted desired digit from scanned and image and then applied re-scalling having size 28 × 28.
• At the end of pre-processing stage, all images are converted into binary form (please see Fig. 2)

In the binarization process object available in the original image is separated from the background region. This process is called thresholding. The thresholding principal is based on the idea that a grayscale image is converted into binary form before further process. The size of the data can be reduced by this way, thereby reducing computational cost. The binarization process separate foreground region from background in a scanned document. Literature reported two kinds of thresholding methods, i.e., global, and local thresholding. In recent days another thresholding method is also used by researchers which is called hybrid approach. Hybrid approach combines both local and global thresholding. We in the proposed work adapted hybrid thresholding. The hybrid approach uses strength of both methods which provides comparatively better mechanism for the system. Some benefits we noticed for hybrid methods during experimentation are less computational cost, good flexibility and better efficiency, robustness, and lastly better accuracy in terms of foreground and background regions extractions.

Images are arranged and named in the format as follows;

An example image with the corresponding name is shown in Fig. The initial digit of the name shows the subject number in the dataset. The second part of the name shows the version of writing. The last part of the name shows the digit number.

## Ethics Statement

Informed consents have been obtained from all particpants who participated in writing these hand written text. A consent form has been signed from each participant while collecting this dataset. It is also worth noting that the images collected do not contain any sensitive information about the subject's identity.

## CRediT Author Statement

**Khalil Khan:** Methodology, Data curation, Writing – original draft; **Rehan Ullah Khan:** Methodology, Investigation, Investigation, Conceptualization, Supervision, Validation, Writing – review & editing.

## Declaration of Competing Interest

The authors declare that they have no known competing financial interests or personal relationships that could have appeared to influence the work reported in this paper.

## Data Availability

Pashtu Language Digits Dataset (Original data) (Mendeley Data).
